# Unravelling the genomic origins of lumpy skin disease virus in recent outbreaks

**DOI:** 10.1186/s12864-024-10061-3

**Published:** 2024-02-19

**Authors:** Priya Yadav, Ankeet Kumar, Sujith S Nath, Yashas Devasurmutt, Geetha Shashidhar, Madhvi Joshi, Apurvasinh Puvar, Sonal Sharma, Janvi Raval, Rameshchandra Pandit, Priyank Chavda, Sudeep Nagaraj, Yogisharadhya Revanaiah, Deepak Patil, S K Raval, Jigar Raval, Amit Kanani, Falguni Thakar, Naveen Kumar, Gundallahalli Bayyappa Manjunatha Reddy, Chaitanya Joshi, Baldev Raj Gulati, Utpal Tatu

**Affiliations:** 1 Department of Biochemistry, Indian Institute of Science, Bangalore, 560012 India; 2 Central Research Laboratory, KIMS, Bangalore, 560070 India; 3 ICAR-National Institute of Veterinary Epidemiology and Disease Informatics, Bangalore, 560064 India; 4 Kamdhenu University, Gandhinagar, Gujarat India; 5 AH Department, GoG, Gujarat India; 6 National Centre for Veterinary Type Cultures, ICAR-NRC on Equines, Sirsa Road, Hisar, Haryana 125001 India; 7 Gujarat Biotechnology Research Centre, Gandhinagar, 382011 India

**Keywords:** Lumpy skin disease virus, Whole genome sequencing, Mutation analysis

## Abstract

Lumpy skin disease virus (LSDV) belongs to the genus *Capripoxvirus* and family *Poxviridae*. LSDV was endemic in most of Africa, the Middle East and Turkey, but since 2015, several outbreaks have been reported in other countries. In this study, we used whole genome sequencing approach to investigate the origin of the outbreak and understand the genomic landscape of the virus. Our study showed that the LSDV strain of 2022 outbreak exhibited many genetic variations compared to the Reference Neethling strain sequence and the previous field strains. A total of 1819 variations were found in 22 genome sequences, which includes 399 extragenic mutations, 153 insertion frameshift mutations, 234 deletion frameshift mutations, 271 Single nucleotide polymorphisms (SNPs) and 762 silent SNPs. Thirty-eight genes have more than 2 variations per gene, and these genes belong to viral-core proteins, viral binding proteins, replication, and RNA polymerase proteins. We highlight the importance of several SNPs in various genes, which may play an essential role in the pathogenesis of LSDV. Phylogenetic analysis performed on all whole genome sequences of LSDV showed two types of variants in India. One group of the variant with fewer mutations was found to lie closer to the LSDV 2019 strain from Ranchi while the other group clustered with previous Russian outbreaks from 2015. Our study highlights the importance of genomic characterization of viral outbreaks to not only monitor the frequency of mutations but also address its role in pathogenesis of LSDV as the outbreak continues.

**Supplementary Information:**

The online version contains supplementary material available at 10.1186/s12864-024-10061-3.

## Background

The Lumpy Skin Disease is caused by Lumpy Skin Disease Virus (LSDV) [1] which is a double-stranded DNA virus of genome size 150 kb and belongs to the genus *Capripoxvirus*, sub-family *Chordopoxviridae* and family *Poxviridae* [2]. The other members of the genus are Goat poxvirus (GTPV) and Sheep poxvirus (SPPV). LSDV is very similar to all the members of the Poxvirdae family in morphological characteristics, such as being very similar to the vaccinia virus under electron microscopy [3]. LSDV is non-zoonotic and is known to infect specific hosts, including cattle (*Bos indicus*, *Bos taurus*) and domestic water buffaloes (*Bubalus bubalis*) [4–6]. In recent years, reports have emerged that LSDV can also infect camel, giraffe, and wildebeest in the wild [7–9]. It spreads via contact through skin lesions, milk, and blood-sucking insects such as biting flies, ticks and mosquitoes [10–13]. The infection spreads more in the warm and wet periods as compared to the winters due to the increased insect population and its mobility in summers [14].

LSD virus was first reported in 2019 from India and has since caused several outbreaks. In the recent outbreak, cases started to appear in May 2022. Around 1 lakh cows have died as per recent reports in the current outbreak [15]. In a developing country like India, livestock production constitutes one of the important ways of earning a livelihood, and a deadly disease such as Lumpy Skin Disease has caused direct loss to the economy and poor production of livestock [15]. India has a cattle population of 308 million; therefore, controlling the spread of infectious diseases is important [16].The direct loss includes deaths of cattle, a decrease in milk production while the indirect losses include movement restriction of cattle across the country [15]. Earlier studies have reported pathological changes in most of the organs and tissues of infected animals such as cow mastitis, necrotic hepatitis, lymphadenitis, orchitis and also in some cases, myocardial damage [17].

World Organization of Animal Health (WOAH) has now identified lumpy skin disease as a notifiable disease [18]. Since its first discovery in Zambia in 1931 [19], this disease was initially confined to the Sub-African region until 1989 and then it started spreading across boundary to Middle East Asia [20, 21]. LSDV was reported in 2016 in Russia and other Southeast European nations [21]. The disease first appeared in India along with other Asian countries such as China, Nepal, Thailand, Bangladesh, Bhutan in November 2019 [22, 23]. While LSD has been reported in India since 2019, it has caused significant damage during 2022 outbreak by infecting more than 2 million cows. The symptoms of LSD vary in individual animals depending on the severity of the infection. An animal takes 1–4 weeks to develop symptoms such as high fever, ocular and nasal discharge, loss of appetite, nodular lesion on skin [24]. According to the data available, a mortality rate of 5–45% was observed [4, 25–27]. The major states affected in terms of mortality and morbidity are Rajasthan, Gujarat, Uttar Pradesh, Punjab, Haryana, Karnataka, West Bengal and Maharashtra [28]. Due to the lack of treatment options, only way of preventing infection is vaccination and by separating the infected animals. The Indian government has taken several measures to control the spread of LSD, including mass vaccination campaigns (goatpox vaccine), setting up quarantine facilities and restricting the movement of infected and susceptible animals. However, the disease continues to be a challenge due to a lack of awareness about its transmission and control, as well as the difficulty in detecting infected animals in the early stages of the disease.

It is generally recognized that the LSDV may have originated from one of the earlier pox virus species and then evolved by spreading in different kind of hosts. Double-stranded DNA viruses are known to use homologous recombination for evolution towards expanding their host range and virulence [29]. In this study, we have utilized genome sequencing to identify the variants of LSDV circulating in India. Through the phylogenetic analysis we found there are two different classes of variants in India. We then performed mutation (SNP) analysis and found the groups differ significantly in the number of mutations.

## Results

### Analysis of LSDV cases in the field reveal varied signs and clinical outcomes

**Fig. 1:**
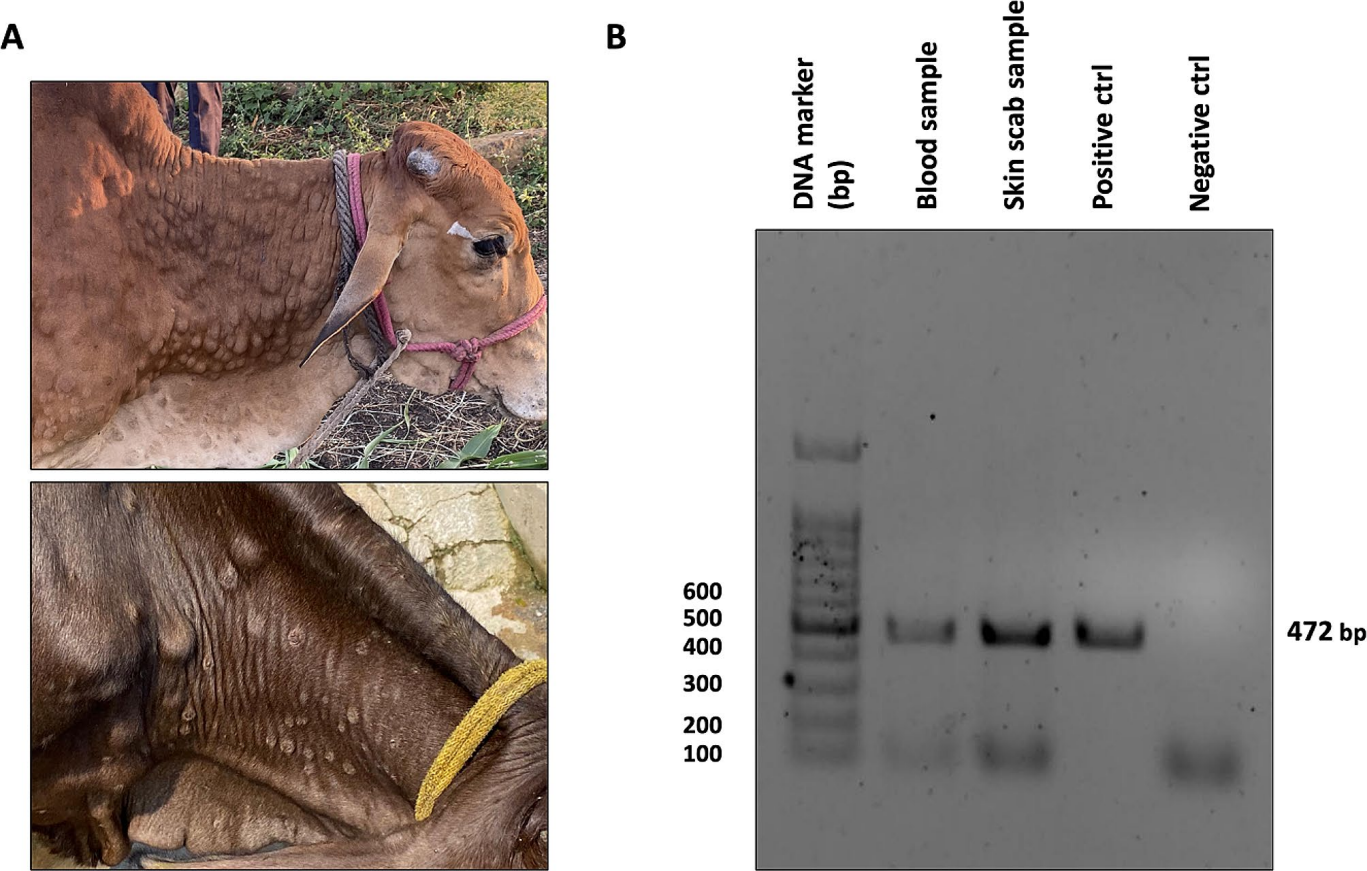
Clinical signs and molecular diagnostics. (**A**) Animal showing severe skin lesions and nodules on body and appearance of swollen lymph nodes (**B**) PCR-based confirmation of LSDV by using partial viral fusion gene amplification

Samples of blood, oral swab and skin scab from infected cows were collected from Karnataka, Maharashtra, Rajasthan, and Jhansi during August-November 2022 while the outbreak was going on in India. The animals were showing typical clinical signs such as reduced milk yield, loss of body mass, raised body temperature upto 40–41 °C, nasal and lachrymal discharge, lost appetite, and skin nodules observed on the body of both young and adult cows. The skin nodules burst open when the viral titre is high, exposing the internal layers of the skin which leads to open wounds and eventually secondary bacterial infection if not timely treated (Figure 1A). The villages observed mortality rate as high as 40% for infected cows, and higher mortality was evidenced in young cows as compared to the adults.

Vectors such as flies, blood-sucking ticks, and mosquitoes were also observed in the vicinity of the animals. Due to the lack of proper ventilation and isolation facilities for cattle, LSDV managed to infect over 20 million cows in 15 different states, and the outbreak is still ongoing.

### PCR-based detection strategy for LSDV

The diagnosis for Lumpy skin disease mainly depends on the typical clinical signs and differential diagnosis. The severe form of the disease has characteristic symptoms while the early signs of LSDV overlaps with other diseases like bovine herpes mammillitis, bovine papular stomatitis and foot and mouth disease [30]. Agar gel precipitation is also used to detect viral antigen in a serum or tissue sample. But this test is not specific and cannot be used for LSDV because the antigens of LSDV are shared with other poxviruses. Therefore, molecular diagnosis by using PCR is the most effective method to detect LSDV.

Samples including blood, oral swab, skin scabs were collected from different parts of India and transported to the ICAR and IISc laboratory to confirm LSDV infection. Conserved regions in the virus genome were identified using multiple sequence alignment and then primers were designed for detecting LSDV. LSDV specific primers were used for fusion gene LSDV0117, which helps the viral envelope fusion with the host membrane as described in method section. Conventional PCR was performed and the amplicon size of 472 bp was confirmed (Fig. 1B), full image (Supplementary Fig. 1). All the samples collected from symptomatic cows were positive for Lumpy Skin Disease Virus.

### Amplification of Indian LSDV strain genome to develop amplicon-based whole genome sequencing

DNA extracted from 22 samples (including twelve skin scab/nodule samples from cows and ten virus samples collected after cell culture passage) was used for further processing. The whole genome of LSDV is approximately 150 kb, so it was necessary to generate smaller amplicons (3 kb) to achieve full genome coverage for Oxford Nanopore Technology and overlapping primers were designed. Nanopore sequencing produces long reads, which are useful for characterizing complex genomic regions.

A total of 1,126,683 reads were obtained, ranging in size from 105 bp to 54,253 bp, with a median size of 10,000 bp. Reads smaller than 1.5 kb were filtered out to avoid non-specific reads. The coding sequences of 156 genes were obtained using GeneMarkS2 for ab initio gene prediction. The genome was assembled from the raw sequencing data, and the location of the genes was identified. Additional 12 samples collected from Jamnagar, Anand, and Surat were sequenced using Illumina sequencing, covering over 90% of the genome sequence.

### Phylogenetic analysis using whole genome sequences of LSDV reveals multiple strains

**Fig. 2:**
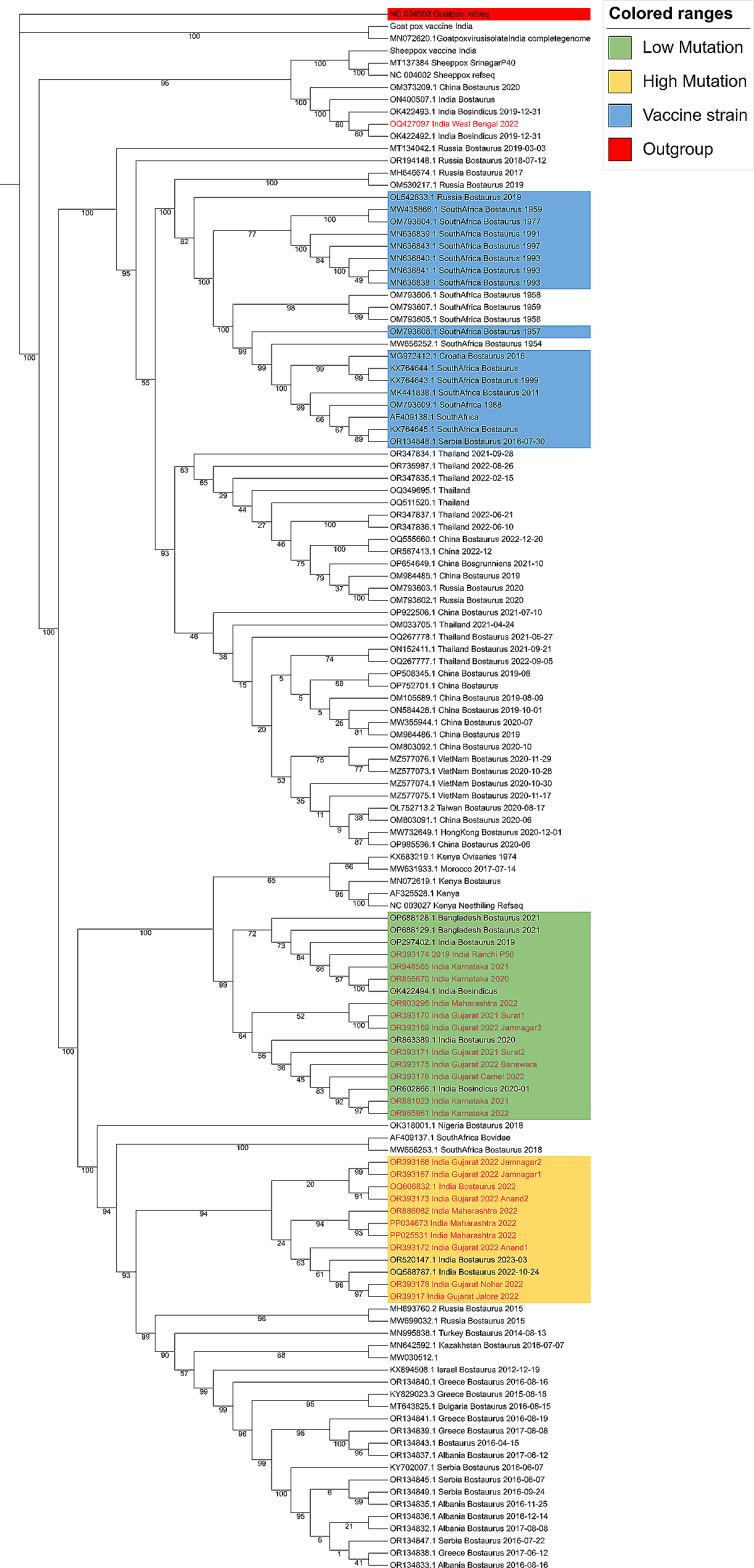
Phylogenetic analysis of LSDV. Maximum-likelihood Bayesian phylogenetic tree based on whole genome sequences showing the relationship in Capripox virus family including Sheep poxvirus, Goat poxvirus and complete genome sequences of LSDV strains sequenced from many parts of India during 2022 outbreak as well as sequences available on NCBI from previous outbreaks. The genomes sequenced in this study are highlighted in red text

Multiple sequence alignment of the LSDV whole genome sequences from different regions of the world was performed and then used to construct a maximum likelihood-based phylogenetic tree, which shows the evolutionary relationships among different LSDV strains and other pox viruses. Goat poxvirus was used as outgroup, as shown in Fig. 2.

The genome sequences from 2022 outbreak (India) lie on two separate branches on the phylogenetic tree, which has been labelled as low-mutation and high-mutation (Fig. 2). Low-mutation genomes closely resemble the Ranchi sequence from 2019 outbreak and Hyderabad sequence from 2020 outbreak. This group has lesser number of variations in their genome sequences as compared to the reference Neethling LSDV sequence. The other group, high-mutation, with higher number of variations per sequence, lies separately, grouping with Russian 2015 LSDV outbreaks. This observation suggests that the current 2022 LSDV outbreak in India is a result of two different group of strains circulating together in the same region.

The Indian LSDV sequences from 2022 are distinct from the vaccine strains of LSDV, confirming that the recent outbreak in India is unlikely to be the result of vaccine spillover. All the Neethling virus-based vaccine strains and vaccine-derived recombinant strains, characterized by combined genetic sequences [31], form a separate group. The close resemblance of samples from recent outbreak to the 2019 Ranchi strain suggests that infections were occurring, although not very severe, in cows for a few years. However, the higher number of mutations in high mutation group indicates the circulation of another strain with an increased mutation rate in genes involved in host cell binding and immune evasion.

The Indian LSDV sequences are 99.98% identical to the neethling strain and 97.38% identical with Goat poxvirus. LSDV is known to code for 156 proteins, and most of the genes are common between these viruses. The differences in LSDV and goatpox sequences lies in the terminal region of the genome. These terminal regions contain genes important for virulence such as ankyrin repeat-domain containing proteins which helps the virus to bind to the host cell.

### SNP analysis in the genome sequence reveals genetic variants with potential to cause severe infection

**Fig. 3:**
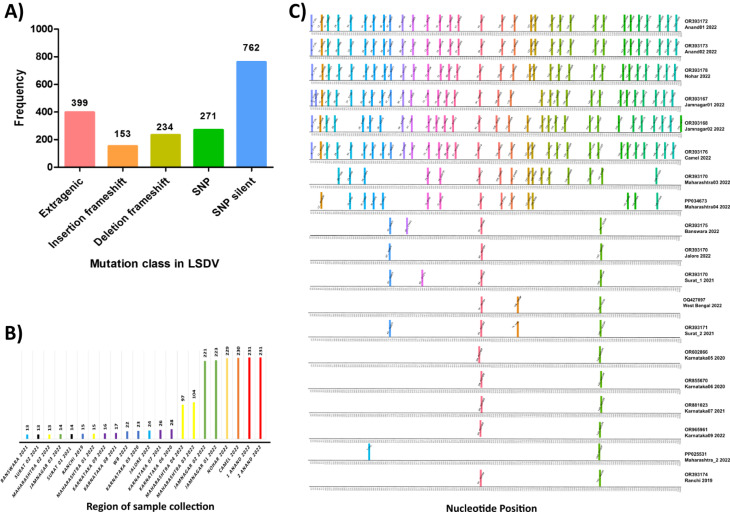
The variations in 22 LSDV genome sequences from India were classified into mutation classes (**A**) All classes of variations present in a total of 22 genomes, most of the variations belong to SNP_Silent followed by extragenic mutations (**B**) Total number of variations identified in LSDV strains from 2022 outbreak. The colors in the graph used are for various regions in India (**C**) Single Nucleotide Polymorphisms (SNPs) on amino acid level across all LSDV genomes sequenced

**Fig. 4:**
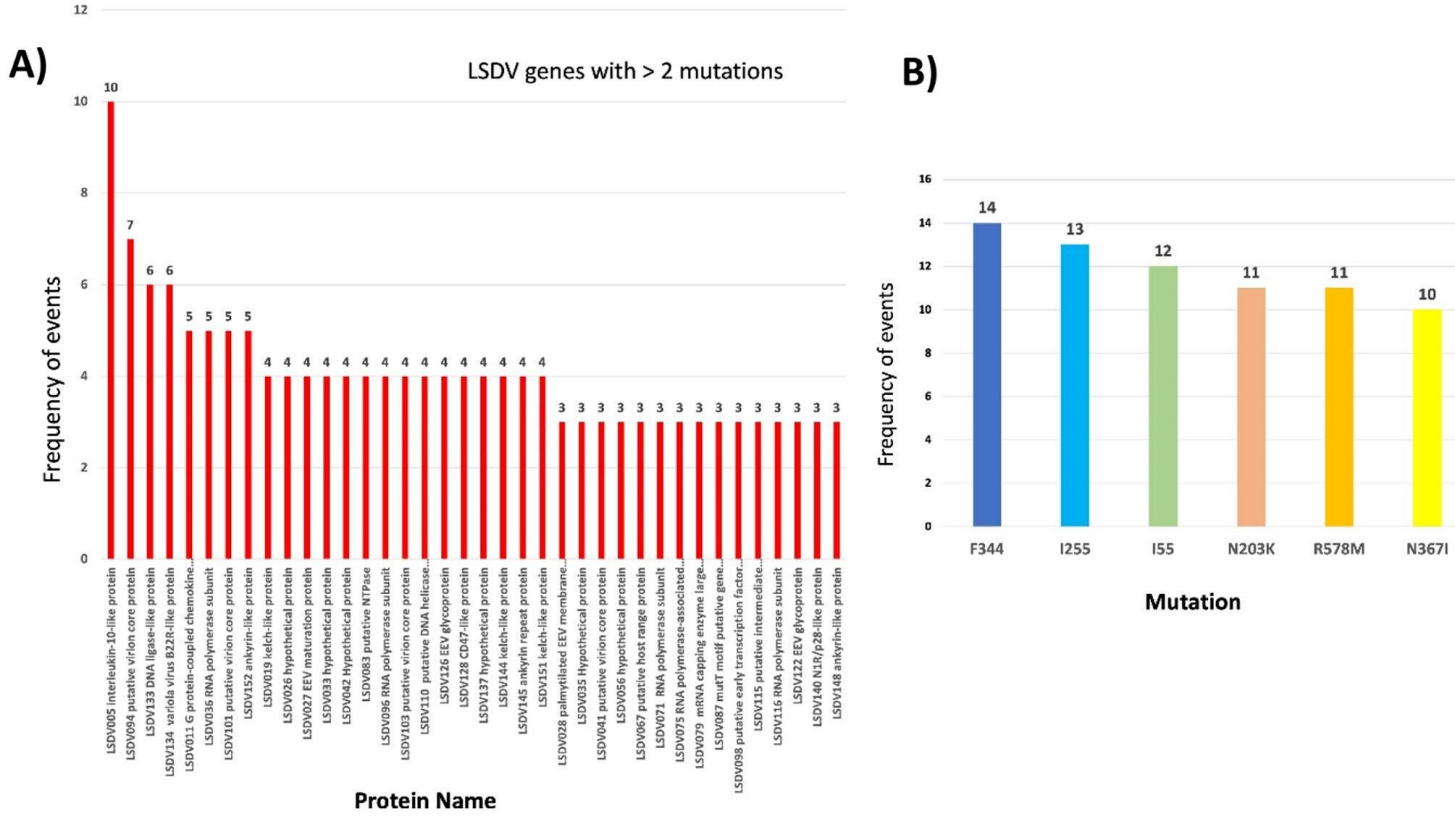
(**A**) Graph showing number of variations in protein-coding genes in LSDV, with more than 2 variations per gene represented. (**B**) The graph represents the most common mutation on amino acid level in 22 LSDV genome sequences upon analysis

We performed mutation analysis using nucmer and found a total of 1819 variations in the 22 sequenced genomes compared to the reference sequence LSDV Neethling strain (NC003027.1). These variations at the amino acid level were dominated by silent SNPs (synonymous), followed by extragenic, SNPs (non-synonymous), deletion frameshift, and insertion frameshift mutations (Fig. 3A). High-mutation group sequences exhibit the highest number of SNPs at the amino acid level (Fig. 3B). Among the LSDV genes, 38 genes have more than two variations per gene, with the gene encoding Interleukin-10-like protein having the highest number of variations [10]. This is followed by the virion core protein gene with 7 variations, DNA ligase-like protein, B22R-like protein, and Ankyrin-like protein with 6 and 5 variations, respectively (Fig. 4). Other genes, such as Kelch-like proteins, RNA polymerase subunit, EV glycoprotein, DNA helicase transcriptional elongation factor, and early transcription factor large subunit, important for virus binding to host cells and virus replication, have three to four variations in each gene. Additionally, 25 genes have two mutations, including several hypothetical protein-coding genes, and 34 genes show one variation only. Twenty out of 22 genomes sequenced in this study showed non-synonymous changes at amino acid level (Fig. 3C).

The most mutated protein-coding gene, namely LSDV005, coding for Interleukin-10-like protein is a functional viral cytokine homolog that plays a role in regulating host immune response, as inferred from UniProt. This protein has 4 helix cytokine-like core. Viral interleukins have been shown to activate cellular signalling cascades that enhance viral replication [32]. Previous gene knockout studies have shown that LSDV005 is one of the important protein-coding genes which is responsible for the virulence of the LSD virus in host cells. LSDV005 is similar to cellular IL-10 in the carboxyl terminus part and affects the immune system similarly [33].

Another highly mutated gene is LSDV094, which codes for virion core protein. LSDV genome consists of 10 virion core protein-coding genes throughout the genome which make up the core/nucleoprotein of the LSD virus. One common SNP in this gene in High-mutation leads to R578M transition, that is from basic to non-polar amino acid. Other variations inlude R581Q. Virion core proteins are one of the largest families of poxvirus proteins, many of which have been correlated as virulence factors [34].

Other genes with major variations include LSDV134, which encodes for variola virus (VV) B22R like protein. It is a VV immunomodulating gene with sequence homology to serine protease inhibitors (serpins) that possess antiapoptotic and anti-inflammatory properties. B22R has been shown to reduce the host’s immune response to the virus and rabbitpox equivalent of VV B22R has been shown to inhibit apoptosis in a caspase-independent manner and increase host range as well [35].

Another important gene which is mutated on protein level is LSDV036 which encode for RNA polymerase subunit. Poxviruses encode a multi-subunit DNA-dependent RNA-polymerase that carries out viral gene expression in host cytoplasm. There were two silent mutations, and 2 SNPs observed in High-mutation genome sequences. The SNP leads to F130S, V297A substitution and given its role in the regulating transcription, any mutations in this gene can lead to altered ability to proliferate and cause symptoms.

Gene LSDV140 encoding for Ring finger host range protein or p28-like protein was also found to be mutated with N203K transition in low-mutation while high-mutation showed SNPs T132M and S152F. P28-like protein is a part of Ub system and acts as Ubiquitin ligase. Similar mutation was reported earlier also in this protein [36]. These findings highlight the importance of consistent monitoring of genetic variations among LSDV variants. Rest of the SNPs belong to hypothetical proteins, whose functions are yet to be identified in poxviruses. Out of 156 genes of LSDV, more than 40 protein coding genes are hypothetical, and their function remains unknown. 26 of these hypothetical protein coding genes were found to have variations. A total of 399 extragenic mutations are present in 22 genome sequences, and most of the variations were observed in 3’UTR. Since 3’UTRs are involved in post-transcriptional regulation, and mRNA stability and degradation, these variations might affect gene expression and regulation in the host cell. Supplementary File 1 presents the number of mutations found in different proteins in 22 genomes reported in this study.

Many unique mutations were also identified in the genomes sequenced in the current study, such as N1 sample collected from Jamnagar, Gujarat in 2022 has K114I in gene LSDV005 coding for Interleukin-10-like protein; West Bengal sample showed unique SNP Y418F in gene LSDV116 coding for RNA polymerase subunit; Banswara, Rajasthan 2021 genomes sequence showed Y194C in LSDV057 coding for putative virion core protein. Mutations in these proteins can be one of the sophisticated mechanisms for enhancing the spread of virus and these mutations can affect the virulence and host range of the virus. These mutations can arise spontaneously or due to selection pressure from the host immune system and from the use of vaccines.

The most common mutations found in Indian LSDV 2022 sequence include changes in the viral envelope protein, which can alter the ability of virus to evade the host immune response, or in the viral polymerase gene, which can affect the replication and spread of the virus. It is important to monitor the genetic variation in LSDV in India, as it can help to understand the evolution and transmission of the virus and to design effective control strategies, including diagnostic tests.

## Discussion

The frequency in the occurrence of the outbreak and spread of the virus from its original geographic range has led to increased research. The first major outbreak in India happened in 2019, most of the sequences deposited for that outbreak are closely related to Neethling reference strain harbouring a few mutations. But the recent outbreak in India shows presence of at least two types of variants circulating in India.

To gain a comprehensive understanding of the variants of LSDV and their circulation patterns, samples were collected from different parts of India. The phylogenetic analysis of these sequences revealed two major groups. Analysis of the sequence composition at the nucleotide and protein levels demonstrated significant differences in the occurrence of single nucleotide polymorphisms (SNPs) between the two groups. The group that is closer to the neethling strain exhibited less variation, while the other group displayed a higher number of mutations, most of which did not result in amino acid changes. Generally, the poxviruses are not fast evolving viruses, such as Variola virus is estimated to be evolving at ˜ 0.9–1.2 × 10^− 6^ substitutions/site/year [37]. But they can undergo mutations due to mechanisms such as homologous and nonhomologous recombination, gene duplications, gene loss, and the acquisition of new genes through horizontal gene transfer.

Recently, Schalkwyk et al. established the substitution rate of 7.4 × 10^− 6^ substitutions/site/year in Lumpy skin disease virus [38]. Therefore, it is possible that the high severity in the recent outbreaks has increased due to the increased number of mutations, as seen in high-mutation, containing sequences from Indian regions with high-severity cases such as Rajasthan, Gujarat, and Maharashtra, which enables the virus to replicate more and cause more clinical symptoms and resulting in high mortality. Similarity in mutations of high-mutation strains with Nigeria 2021 and Russian 2015 outbreak suggests that there might have been a transboundary migration of an infected animal as both groups show higher number of similar mutations. Some SNPs caused changes in amino acids within proteins, particularly in genes like Interleukin 10-like protein, virion core-like protein, and GPCR-like protein. These mutations could be due to the evolutionary pressure on the virus resulting from the widespread use of vaccines that are not very effective, leading to mutations in important viral immune response genes.

Field analysis of LSD cases revealed a wide range of clinical signs and outcomes in infected cattle, including reduced milk production, weight loss, elevated body temperature, nasal and lachrymal discharge, loss of appetite, and the development of skin nodules. When these nodules burst open, they can lead to open wounds and bacterial infections. The high mortality rate, especially among young cattle, highlights the severity of LSDV infection. The increased severity observed in samples from Gujarat, Maharashtra, and Rajasthan may be a result of the high number of variations in those regions. Analysis of LSDV genomes identified numerous genetic variations, including silent SNPs, extragenic mutations, deletion and insertion frameshift mutations, and SNPs leading to amino acid changes. Several genes with multiple variations were found, including those encoding Interleukin-10-like protein, virion core protein, RNA polymerase subunit, and B22R-like protein. These variations can influence virulence and host range of the virus. Recently, there have been reports of LSDV infection in yaks [39], camels, free ranging gazelles in India, and giraffes in Vietnam [8, 9, 40], indicating that LSDV can undergo transmission across species with an increased number of mutations. A total of 230 variations were observed in the LSDV strain sequenced from camel hosts. These SNPs were similar to the strains in High-mutation sequenced from cow hosts. Mutations in proteins like B22R, coded by LSDV134, which have been shown to reduce the host’s immune response to the virus and increase host range, were also found in the LSDV strain from camels [35]. Therefore, it becomes important to identify and vaccinate other hosts which can act as reservoirs for the virus. Monitoring genetic variations in LSDV is crucial for understanding its evolution, developing control strategies, and detecting new outbreaks. The easy spread of LSDV through direct contact, vectors, and bodily fluids emphasizes the importance of proper animal management and biosecurity measures to prevent and control the disease.

Sheep poxvirus and Goat poxvirus, both belonging to the genus *Capripoxvirus*, have been endemic in India. However, LSDV outbreaks only started recently in Southeast Asian countries in 2019 [41]. Another poxvirus of the *Poxviridae* family, Smallpox virus, which caused significant damage to human health and life, has been globally eradicated in the last century through large-scale vaccination campaigns using live vaccinia vaccines [42]. Therefore, most effective way to prevent and eliminate disease is vaccination. While there are vaccines for LSD based on the Neethling strain. However, animals sometimes develop clinical symptoms such as the formation of nodules and a drop in milk yield even after vaccination. This adverse effect is known as ‘Neethling disease’ [43]. The commonly used Goat poxvirus vaccine is often employed to prevent LSDV, due to its antigenic similarity to LSDV. The fact that some outbreaks are occurring in vaccinated animals raises about the complete effectiveness of the vaccine. Possible reasons for this include the vaccination method being ineffective, flaws in the administration methods, or issues with vaccine storage.

Some studies report that the commonly used LSDV vaccine is not a pure viral culture of LSDV virus but instead contains a mix of quasi-species, which raises the chances of homologous recombination in the genome of viruses [31]. It has been reported that LSDV can also undergo recombination [29]. Therefore, while using live attenuated viruses, it is important to analyze the quality and nature of variation. Previously, several vaccine-like recombinant LSDV strains were discovered in Kazakhstan, neighbouring of Russia and China from 2017 to 2019 [44]. In our study, we analyzed the current LSDV outbreak clusters and observed that both the groups lie away from the vaccine strains as well as the recombination-derived strains. Therefore, the current outbreak is not a result of vaccine spillover. The 22 LSDV genomes sequenced in this study are separate from the recombinant LSDV strains from Southeast Asia and the Russian vaccine spillover LSDV strains, as clearly depicted in the phylogenetic analysis.

A Ranchi strain-based homogenous live-attenuated LSD vaccine, Lumpi-Pro VacInd (an experimental vaccine) has been developed to prevent the outbreaks. The ‘Neethling disease’ event in which animals develop a reaction against vaccination such as skin nodule formation was not observed in field animals and the vaccine was shown to be 100% effective till January 2023 [45]. The SNP analysis of this vaccine strain showed that it belongs to the LSDV sequences of Low-mutation in the phylogram which has lesser number of mutations. It has been shown that it neutralizes LSDV strains from 2022 outbreak [45]. Continued monitoring of vaccinated animals for extended periods will be important to assess the vaccine’s efficacy.

To prevent future outbreaks of LSDV, it is crucial to identify the possible reasons behind these outbreaks and strategize accordingly. It is important to identify all potential hosts for LSDV beyond cattle and arthropod vectors. Regulating the transboundary migrations of animals is necessary to prevent all possible pathways for LSDV introduction. Regarding vaccines, live attenuated LSDV strain vaccines should only be used after undergoing quality testing.

## Conclusion

The analysis of LSDV cases in India during the 2022 outbreak highlighted the severe impact of the disease on cows and the significant economic losses incurred in the agricultural sector. Developing a molecular detection strategy using PCR allowed for accurate diagnosis of LSDV, overcoming the challenges posed by clinical signs overlapping with other diseases. Whole genome sequencing provided valuable information on the origin and evolution of the LSDV strains circulating in India. The phylogenetic analysis revealed the presence of two distinct groups, suggesting multiple introductions of the virus. SNP analysis identified genetic variations in essential genes, potentially affecting virulence and antigenicity.

These findings contribute to a better understanding of LSDV and provide valuable information for developing effective control strategies, such as development of diagnostic tests. Monitoring genetic variations and protein expression patterns is crucial for detecting new outbreaks, tracking the virus’s evolution, and guiding the development of targeted interventions. Overall, this research enhances our knowledge of LSDV and its impact on cow’s health, supporting efforts to mitigate its spread and minimize economic losses in the future.

## Material and method

### Sample collection

Biological specimens including saliva swab, blood sample, skin lesion scabs were collected from infected cattle as per the standard practices without using any anaesthesia by veterinarians in Karnataka in India. An informed consent was taken from the cows owners before collection of samples. The animals presented symptoms such as fever, nasal discharge and characteristic pox nodular skin lesions. Blood samples were collected in EDTA vacutainers (Becton-Dickenson) and the skin lesions were collected and transported to the laboratory in 50% glycerol (Qualigens). All samples from the previous outbreaks (2019–2021) were available as cell cultures. For the 2022 samples, some were directly processed for DNA extraction, while others were first propagated in either Vero or Lamb testis cell cultures before proceeding with DNA extraction. The samples were used to extract DNA and remaining samples were stored in -80 °C for further use.

### Processing of samples

For molecular diagnosis, 200 µl of Blood sample was used to extract DNA by using Nucleic acid extraction kit (HL-NA-100) from Huwel, and the DNA concentration was estimated using a nanophotometer (Implen).

A total of 22 samples of LSDV were used for sequencing (Table 1). Seven samples were from the outbreaks during 2020–2021, 1 sample from Ranchi outbreak in 2019, and 14 samples were from 2022 outbreak in different parts of India, as mentioned in the table below. Out of 22 samples, 12 were taken from skin nodules from cattle and 10 were taken from cell-infected viral cultures [39].

**Table 1:** Sample metadata for the 22 LSDV genomes sequenced in the study

Sl. No.	State	District	Year	Species	Age	Source of virus	Sample ID
1.	Ranchi	Ranchi	2019	Cow	Adult	Cell infected culture (Vero Cells)	Ranchi 2019 L1 (OR393174)
2.	Rajasthan	Banswara	2021	Cow	Adult	Cell infected culture (Primary lamb testicle cells)	Banswara 2021 L2 (OR393175)
3.	Gujarat	Surat	2021	Cow	Adult	Scab	1 Surat 2021 N6 (OR393170)
4.	Gujarat	Surat	2021	Cow	Adult	Scab	2 Surat 2021 N7 (OR393171)
5.	Gujarat	Jamnagar	2022	Cow	Adult	Scab	1 Jamnagar 2022 N1 (OR393167)
6.	Gujarat	Jamnagar	2022	Cow	Adult	Scab	2 Jamnagar 2022 N2 (OR393168)
7.	Gujarat	Jamnagar	2022	Cow	Adult	Scab	3 Jamnagar 2022 N5 (OR393169)
8.	Gujarat	Anand	2022	Cow	Adult	Scab	1 Anand 2022 N8 (OR393172)
9.	Gujarat	Anand	2022	Cow	Adult	Scab	2 Anand 2022 N9 (OR393173)
10.	Rajasthan	Bikaner	2022	Camel	Adult	Cell infected culture (Primary lamb testicle cells)	Camel 2022 L3 (OR393176)
11.	Rajasthan	Jalore	2022	Cow	Adult	Cell infected culture (Primary lamb testicle cells)	Jalore 2022 L4 (OR393177)
12.	Rajasthan	Nohar	2022	Cow	Adult	Cell infected culture (Primary lamb testicle cells)	Nohar 2022 L5 (OR393178)
13.	Maharashtra		2022	Cow	Adult	Scab	MH01 2022 (OR903295)
14.	Maharashtra		2022	Cow	Adult	Scab	MH02 2022 (PP025531)
15.	Maharashtra		2022	Cow	Adult	Scab	MH03 2022 (OR886082)
16.	Maharashtra		2022	Cow	Adult	Scab	MH04 2022 (PP034673)
17.	Karnataka	Raichur	2020	Cow	Adult	Cell infected culture (Vero cells)	KA05 2020 (OR602866)
18.	Karnataka	Bengaluru	2020	Cow	Adult	Cell infected culture (Vero cells)	KA06 2020 (OR855670)
19.	Karnataka	Ramanagara	2021	Cow	Adult	Cell infected culture (Vero cells)	KA07 2021 (OR881023)
20.	Karnataka	Haveri	2021	Cow	Adult	Cell infected culture (Vero cells)	KA08 2021 (OR948565)
21.	Karnataka	Maddur	2022	Cow	Adult	Cell infected culture (Vero cells)	KA09 2022 (OR965961)
22.	West Bengal		2022	Cow	Adult	Scab	WB 2022 (OQ427097)

### Molecular diagnostics

The primers were designed for the viral Fusion gene to confirm LSDV in the collected samples, forward primer- 5’ ATGGACAGAGCTTTATCA, reverse primer- 5’ TCATAGTGTTGTACTTCG (10pM/µl) with Tm 55 °C. The conventional PCR was carried out in 25 µl reaction with the following conditions used: 94 °C for 5 min for initial denaturation, 30 cycles for 94 °C for 1 min denaturation, 55 °C for 30 s annealing, 72 °C for 45 s extension and a final extension step of 72 °C for 5 min. The PCR result was visualized on 1% agarose (Himedia) gel having Ethidium Bromide (Amresco, Cole-Parmer) and analyzed against 100 bp DNA ladder (G-Biosciences) [46].

### Individual PCR for sequencing

A total of 55 overlapping primer sets for 3 kbp amplicons with Tm 66 °C were designed by using PrimalScheme [47], a tool which is used to design primers for multiplex PCR, especially for viral outbreak strains. The complete genome sequence of LSDV from previous outbreak with GenBank ID: OK422493.1 was used to design primers. The DNA extracted from the skin scab sample collected from India was used to amplify the LSDV DNA. The 2-step PCR was performed using Phusion polymerase (Thermofisher Scientific) with the following conditions: 98 °C for initial denaturation, 98 °C for denaturation, 66 °C for annealing and extension, and 72 °C for 3 min for the final extension step. The PCR results were analyzed on 1% agarose (Himedia) gel having Ethidium Bromide (Amresco, Cole-Parmer) and analyzed against 1 kb plus DNA ladder (G-Biosciences).

### Sequencing using Oxford-nanopore technology

The amplified LSDV DNA amplicons of 3 kbp size were pooled together, and the DNA concentration was determined by using a Qubit 3 fluorometer (Invitrogen) after calibrating with the standards, provided by manufacturer. The samples were processed for genome sequencing on MinION (Oxford Nanopore Technologies, Oxford, United Kingdom) with the ligation sequencing kit SQK-LSK109 Oxford Nanopore Technologies (ONT). Samples were sequenced on flow cell R10.3 version (FLO-MIN111) with 1570 active pores. For the MinION, the Barcode 01–09 from the native barcoding kit (ONT) were used. The DNA was cleaned up using KAPA Hyperpure beads (Roche) after every step during sample preparation, and DNA concentration was recorded. The DNA library was prepared as per the ONT protocol by adopting the following steps: end prep of the LSDV amplicons followed by Barcoding and adapter ligation. The flow cell was primed by using the EXP-FLP002 Flow cell priming kit (ONT). Around 300 fmol of DNA library was loaded along with the sequencing buffer and loading beads on the Spot ON MinION sequencer and was run for 11 h on R10.3 flow cell. Base calling and demultiplexing was performed by using Guppy v5.0 command-line software. The reads were aligned to the LSDV whole genome sequence GenBank ID: OK422493 (Ranchi, India, 2019). The alignment-based consensus was generated using a protocol adapted using Artic pipeline v1.2.1 developed for SARS-COV-2, and the gaps were filled by sequencing missing regions using same protocol.

### Sequencing using Illumina NGS technology

NGS library preparation for LSDV whole genome sequencing was done using an in-solution tagmentation-based Illumina Nextera XT DNA Library Prep kit (Illumina) with starting DNA concentration of 1 ng/µl. Prepared libraries were quantified using Qubit 1X ds DNA HS kit (Invitrogen) and the quality check was done on Agilent 2100 bioanalyzer using High sensitivity DNA reagent kit (Agilent). All the libraries were normalized up to 4 nM and pooled. The equimolarized pool of library was processed for the denaturation with 1% PhiX library spike-in as the control library (Illumina). Sequencing was performed on Illumina NovaSeq 6000 SP Reagent Kit v1.5 (300 cycles) paired-end chemistry. Isolated Viral DNA library was sequenced on Illumina MiSeq using MiSeq reagent kit v2 (500 Cycles). The data generated was subject to reference-based assembly using CLC Genomics Workbench v22.0.2 against LSDV reference sequence (NC003027.1). The consensus of each sample were obtained using an inbuilt consensus sequence caller in CLC Genomics Workbench. The variants were visualized using IGV v 2.4.14.

### Phylogenetic analysis and SNP analysis

The newly generated consensus sequence was used along with other LSDV complete genome sequences retrieved from the public domain database NCBI virus, accessed on Dec 25, 2023. The sequences used for phylogeny were aligned using Mafft v7.49, and a maximum likelihood phylogenetic tree was constructed using Iqtree2 version 2.0.7 with 1000 bootstrap value. The tree was visualized using interactive Tree Of Life (iTOL) v6.8.1. Nucmer v3.1 was used to generate SNPs in comparison to the Reference sequence of LSDV (NC003027.1). Nucmer does pairwise alignment to identify SNPs in query sequences compared to reference genome sequence. The table with SNPs was further analyzed using R script in R v4.3.1. The script [48] is available on GitHub (R script link Github).

### Electronic supplementary material

Below is the link to the electronic supplementary material.

**Supplementary File 1.** SNPs in different proteins of 22 genomes of LSDV reported in the study

**Supplementary Fig. 1.** Complete agarose-gel image for PCR confirmation of LSDV in clinical samples (Figure 1B)

## Data Availability

The data that supports the findings of this study is available in the NCBI (BioProject ID PRJNA998208 and PRJNA1004082).
