# Supplementary figures and images for: Unravelling the genomic origins of lumpy skin disease virus in recent outbreaks
Source: BMC Genomics. 2024 Feb 19;25:196. doi: 10.1186/s12864-024-10061-3 (PMC10875885; doi:10.1186/s12864-024-10061-3)

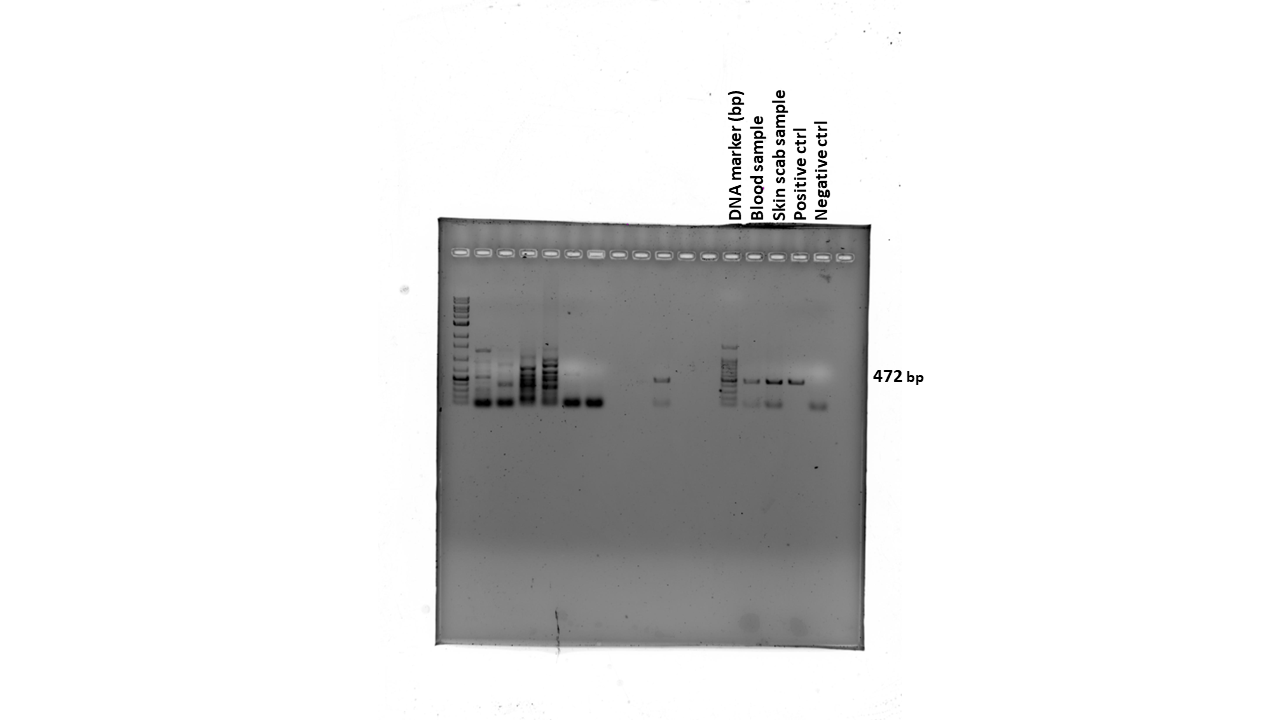

Supplement: Supplementary file 2 — Supplementary Fig. 1. Complete agarose-gel image for PCR confirmation of LSDV in clinical samples (Figure 1B) [file 12864_2024_10061_MOESM2_ESM.tif]
